# Mapping the ‘Two-component system’ network in rice

**DOI:** 10.1038/s41598-017-08076-w

**Published:** 2017-08-24

**Authors:** Ashutosh Sharan, Praveen Soni, Ramsong Chantre Nongpiur, Sneh L. Singla-Pareek, Ashwani Pareek

**Affiliations:** 1 Stress Physiology and Molecular Biology Laboratory, School of Life Sciences, Jawaharlal Nehru University, New Delhi, India; 2 Plant Stress Biology, International Centre for Genetic Engineering and Biotechnology, New Delhi, India

## Abstract

Two-component system (TCS) in plants is a histidine to aspartate phosphorelay based signaling system. Rice genome has multifarious TCS signaling machinery comprising of 11 histidine kinases (OsHKs), 5 histidine phosphotransferases (OsHPTs) and 36 response regulators (OsRRs). However, how these TCS members interact with each other and comprehend diverse signaling cascades remains unmapped. Using a highly stringent yeast two-hybrid (Y2H) platform and extensive *in planta* bimolecular fluorescence complementation (BiFC) assays, distinct arrays of interaction between various TCS proteins have been identified in the present study. Based on these results, an interactome map of TCS proteins has been assembled. This map clearly shows a cross talk in signaling, mediated by different sensory OsHKs. It also highlights OsHPTs as the interaction hubs, which interact with OsRRs, mostly in a redundant fashion. Remarkably, interactions between type-A and type-B OsRRs have also been revealed for the first time. These observations suggest that feedback regulation by type-A OsRRs may also be mediated by interference in signaling at the level of type-B OsRRs, in addition to OsHPTs, as known previously. The interactome map presented here provides a starting point for in-depth molecular investigations for signal(s) transmitted by various TCS modules into diverse biological processes.

## Introduction

**Figure 1:**
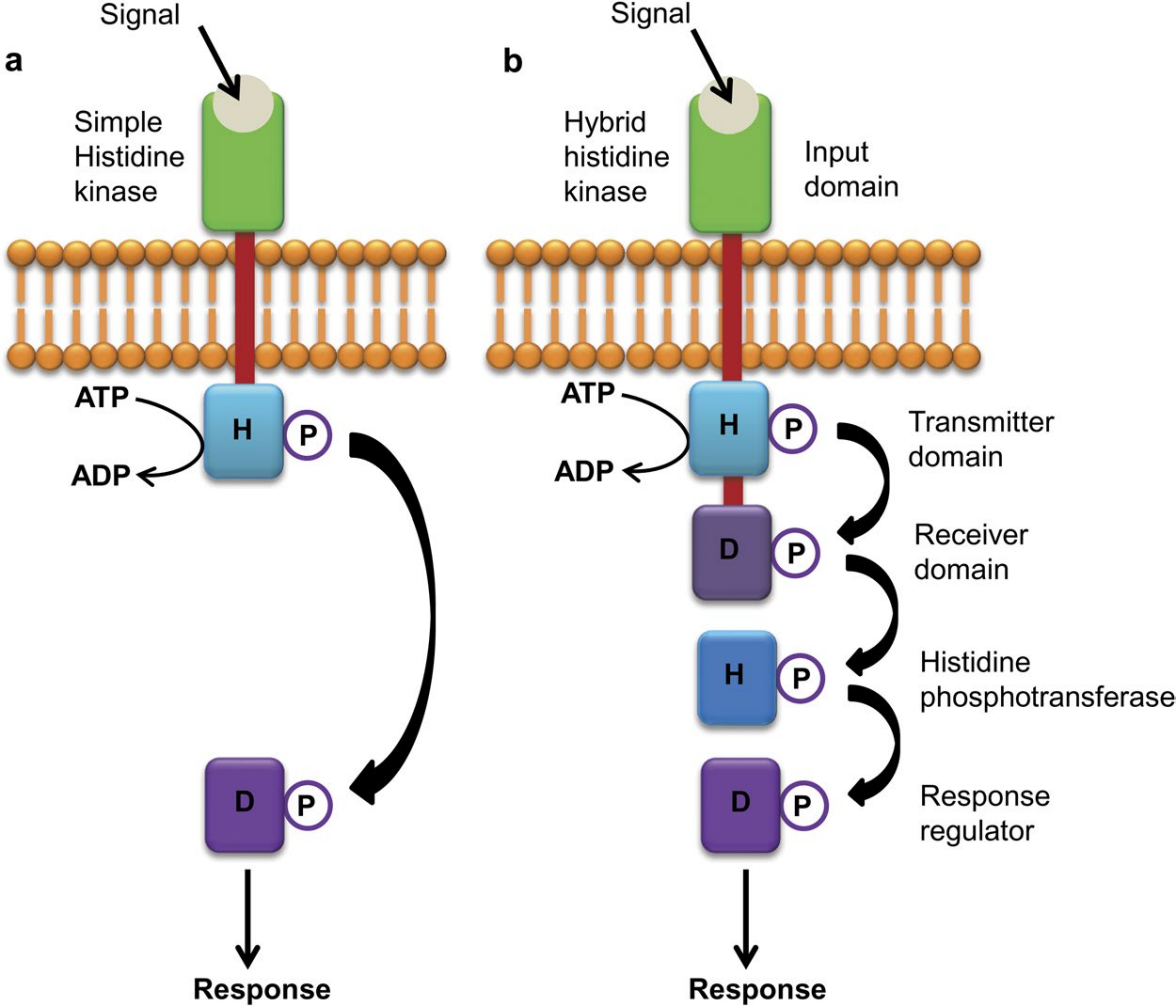
Cartoon depicting the two types of two-component systems along with their component proteins and domains. (**a**) A simple TCS signaling in which signal is perceived by the input domain of a sensory histidine kinase which undergoes auto-phosphorylation at the conserved histidine residue in its transmitter domain. Phosphoryl group is then transferred to conserved aspartate residue, present in response regulator, which regulates the signal response; (**b**) Hybrid-type TCS signaling in which the conserved histidine and aspartate residues are found in the same sensory histidine kinase. An intermediate, histidine containing phosphotransferase protein acts as a mediator for the transfer of the phosphoryl group between the histidine kinase and the response regulator. Arrows indicate transfer of phosphoryl group during phosphorylation events. H, Histidine; D, Aspartate; P, phosphoryl group.

Signaling cascades in living organisms not only enable them to respond appropriately to specific signals but are also a decisive factor for survival under a set of given conditions. Protein phosphorylation is one of the main approaches by which intracellular signaling takes place. Protein kinases carry out phosphorylation of their substrates using ATP as the phosphate donor. Based on the specific acceptor amino acids, they have been categorized into five groups: serine-threonine kinases (STK); tyrosine kinases (TK); histidine kinases (HK); cysteine kinases (CK); and aspartyl or glutamyl kinases (AK)^1^. HKs are operative via two component system (TCS), which are signal transduction pathways that have been found to regulate multiple processes ranging from chemotaxis and nutrient sensing in bacteria to hormone signaling in plants^2, 3^. In simple or prototypical TCS, found exclusively in prokaryotes, signal is perceived by a histidine kinase (HK) and signal transduction occurs via transfer of the phosphoryl group to another group of signal transducer called response regulator (RR) (Fig. 1a). Owing to its importance in sensing of diverse signals, different components and functions of simple prokaryotic TCS have been studied extensively^3–5^^.^

The second type of two-component signaling, found in lower eukaryotic organisms and plants as well, comprises a more complex sensory histidine kinase (hybrid histidine kinase), which contains an extended C-terminal domain with a conserved aspartate residue. In this case, phosphotransfer occurs from the conserved histidine to the conserved aspartate residue, present within the same sensory protein (Fig. 1b). However, the phosphotransfer to the response regulator is mediated by a third class of protein; histidine phosphotransferase (HPT), which itself has a conserved histidine phosphorylation site. After being phosphorylated, the HPT moves to the nucleus and phosphorylates the RR proteins, which in turn, binds to promoters of their target genes and initiate transcription^6–9^. The *Arabidopsis* genome encodes 11 histidine kinases, 5 HPTs and 23 response regulators (ARRs)^6^. Apart from cytokinin signaling, they are involved in various other vital processes such as ethylene signaling, osmosensing, mega-gametophyte development and cold perception^10–17^. More recently, TCS have also been shown to be regulating salt sensitivity, resistance against bacterial and fungal infection as well as diurnal rhythms^18, 19^. Genome wide analysis has revealed the presence of complex TCS machinery in rice, maize, soybean, lotus and populus^20–24^. Though huge diversity has been reported regarding their structure, cellular localization and expression patterns, some of the HKs are yet to be assigned any function^20, 25–29^.

TCS signaling machinery in rice is highly complex, comprising of 11 histidine kinases (OsHKs), 5 histidine phosphotransferases (OsHPTs) and 36 response regulators (OsRRs)^20^. Among the 5 OsHPTs, 2 contain the conserved histidine residue required for phosphorelay activity and are known as authentic phosphotransfer proteins (OsAHP1-2). While the remaining 3 are pseudo-phosphotransfer proteins (OsPHP1-3) which lack the histidine phosphorylation site. The response regulators have been categorized into four groups on the basis of phylogenetic analysis and domain structure: type-A, type-B, type-C and pseudo-response regulators^30^. The type-A response regulators contain the receiver domain and are the primary transcriptional targets of cytokinin signaling^10, 31, 32^. The type-B response regulators contain a Myb-like DNA-binding domain at C-terminal in addition to the receiver domain and act as positive transcriptional regulators of cytokinin signaling^9, 33–36^. The type-C response regulators are phylogenetically more related to the type-A response regulators on the basis of receiver domain sequences and lack DNA binding sequences^37, 38^. The pseudo-response regulators contain a unique CCT domain and play an important role in controlling circadian rhythms. They lack the conserved aspartate phosphorylation site in the receiver domain^30^.

To understand the complexity of rice TCS machinery, we have made an attempt to unravel all possible interactions of proteins within the family. This study was designed to explore the flow of signals as perceived by the sensory histidine kinases and transmitted downstream to different members of this signaling pathway. We found many novel interactions between TCS members, which indicate redundancy in the TCS signaling pathway in rice. Heterologous yeast two-hybrid (Y2H) system has been used to carry out the large-scale analysis of these interactions, followed by reconfirmation *in planta* by bimolecular fluorescence complementation (BiFC) assays. Our interactome data is quite robust and implications of these findings are discussed.

## Results

### Untying the interactions between TCS proteins employing yeast two-hybrid (Y2H) system

Bait and prey constructs of TCS genes were prepared to check protein-protein interactions by Y2H assays (Table 1). Each construct containing a unique TCS gene was transformed into AH109 strain of yeast and transformants were selected on single drop out (SD-Trp/Leu) medium. Thereafter, self-activation of each construct was checked by growth assay on double (SD-Trp/Leu-His + 5 mM 3-AT) and triple (SD-Trp/Leu-His-Ade) drop out medium (Supplementary Fig. S1). None of the prey constructs were found to self-activate the reporter genes *HIS3* and *ADE2* (Supplementary Fig. S1a). However, two of the OsHPTs i.e. OsAHP1, OsAHP2 and all the type-B OsRRs i.e. OsRR22, OsRR23, OsRR24, OsRR26, OsRR27 and OsRR33 (except OsRR21) in bait construct showed self-activation (Supplementary Fig. S1b). These bait constructs, showing self-activation, were not used further for Y2H analysis (Table 1).

**Table 1:** List of different TCS members belonging to various sub-categories, which were successfully cloned and used for Y2H assays (The nomenclature of TCS members is based on Schaller *et al*.^30^).

	Non-ethylene histidine kinases	Phospho-transferases	Type-A Response Regulators	Type-B Response Regulators	Pseudo-Response Regulators
Total members	HK1 HK2 HK3 HK4 HK5 HK6	AHP1 AHP2 PHP1 PHP2 PHP3	RR1 RR2 RR3 RR4 RR5 RR6 RR7 RR8 RR9 RR10 RR11 RR12 RR13	RR21 RR22 RR23 RR24 RR25 RR26 RR27 RR28 RR29 RR30 RR31 RR32 RR33	PRR1 PRR73 PRR37 PRR59 PRR95
Members successfully cloned	HK3 HK4 HK5	AHP1 AHP2 PHP1 PHP2 PHP3	RR1 RR2 RR3 RR4 RR5 RR6 RR9 RR10 RR12 RR13	RR21 RR22 RR23 RR24 RR26 RR27 RR33	PRR1 PRR73 PRR37
Members showed auto-activation in bait vector		AHP1 AHP2		RR22 RR23 RR24 RR26 RR27 RR33	

Note- Those TCS members which showed auto-activation were used only as prey while remaining ones were used as both bait and prey in Y2H assay.

### Determining the interactions between OsHKs and OsHPTs

**Figure 2:**
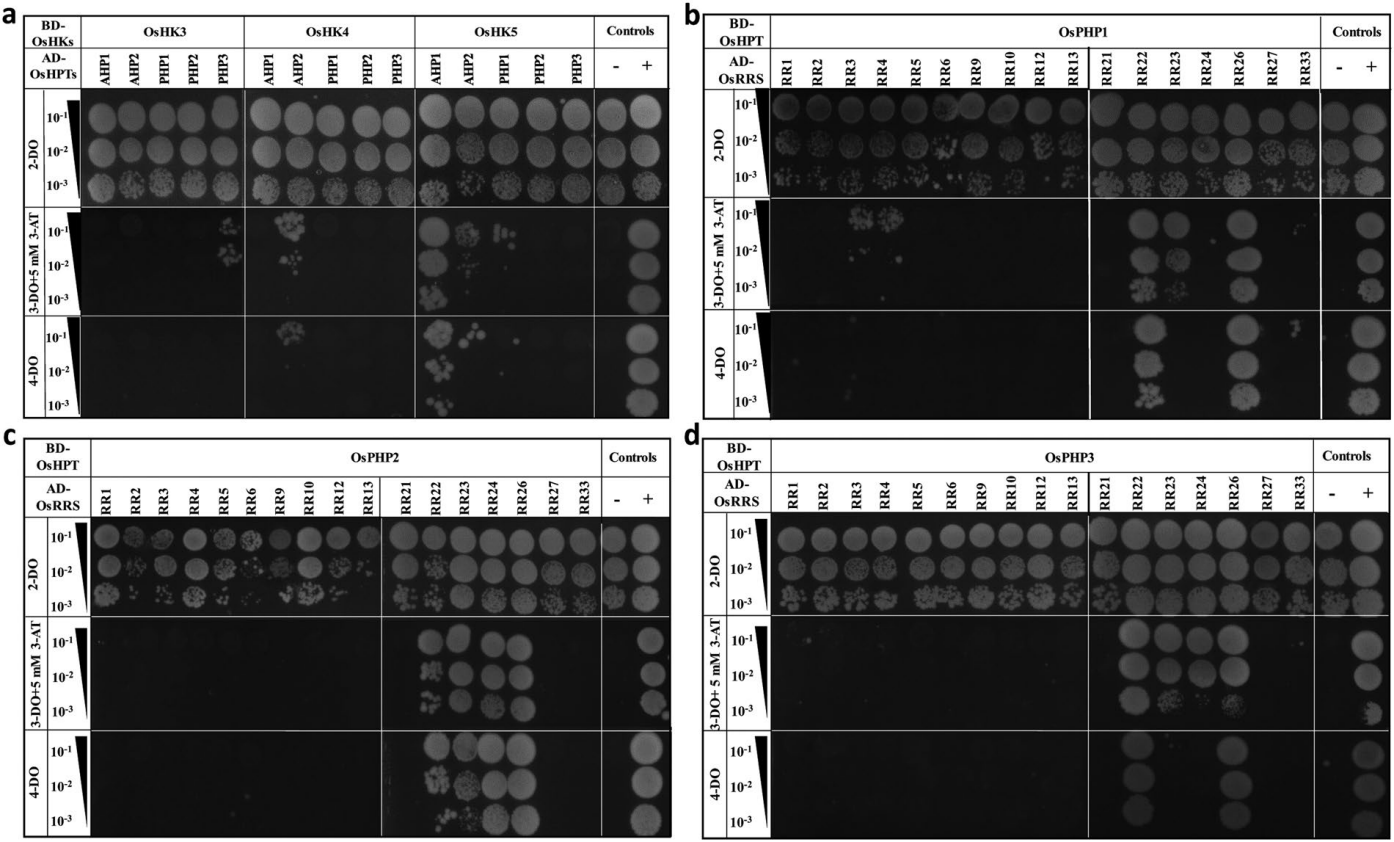
Protein–protein interaction studies among the members of two-component signaling system in rice. (**a**) Y2H analysis for BD-OsHKs-AD-OsHPTs, (**b**–**d**) Y2H analysis for BD-OsHPTs-AD-OsRRs. These interactions were determined using combinations of bait and prey constructs which were co-transformed into yeast. Transformants were checked for *HIS3* and *ADE2* reporter gene activation through serial dilution assays. For this, transformants were spotted on double drop-out medium (2-DO) for growth control, triple drop-out medium (3-DO supplemented with 5 mM 3-AT) to check the activation of *HIS3* reporter gene and on quadruple drop-out medium (4-DO) to check activation of *ADE2* reporter gene. Growth on synthetically deficient triple-drop out and quadruple drop-out media indicates interaction. 10^−1^, 10^−2^ and 10^−3^ represents 10, 100 and 1,000-fold dilutions of cultures of yeast double transformants respectively. “−” and “+” signs represent negative control (host cells co-transformed with empty vectors) and positive control taken as OsSRO1a-pGAD-C1 + OsSOS1-pGBD-C1 respectively. Combinations of bait and prey constructs of TCS members co-transformed into yeast have been mentioned above the serial dilution BD-bait; AD-prey.

For dissecting out OsHKs-OsHPTs interactions, combinations of OsHKs-bait and OsHPTs-prey constructs were co-transformed into yeast. Based on the growth patterns of yeast cells on various selective media, we observed specific interactions between them (Fig. 2). It was observed that OsHK3 interacts with OsPHP3 (Fig. 2a). OsHK4 was found to interact strongly with OsAHP2 as indicated by growth of yeast transformants on triple and quadruple drop-out medium (Fig. 2a). OsHK5 showed interactions with OsAHP1, OsAHP2 and OsPHP1 (Fig. 2a). Interaction with OsAHP1 was strong while those with OsAHP2 and OsPHP1 were weak.

### Determining the interactions between OsHPTs and OsRRs

Similarly, to determine interactions between OsHPTs and OsRRs, combinations of OsHPTs-bait (except OsAHP1-2) and OsRRs-prey constructs were used. Growth assay on triple and quadruple drop-out medium showed multiple interactions of OsHPTs (Fig. 2b–d). We observed the interaction of OsPHP1 with type-A OsRRs- OsRR3 and OsRR4, although the interactions were weak (Fig. 2b). OsPHP1 also showed interaction with type-B OsRRs–OsRR22, OsRR23 and OsRR26. Interactions with OsRR22 and OsRR26 were very strong (Fig. 2b). OsPHP2 did not show interaction with any type-A response regulator but its strong interactions were detected with type-B OsRRs-OsRR22, OsRR23, OsRR24 and OsRR26 (Fig. 2c). Similarly, OsPHP3 did not exhibit interactions with type-A OsRRs but interacted with OsRR22, OsRR23, OsRR24 and OsRR26. Interactions with OsRR23, OsRR24 were of low strength as compared to those with OsRR22, OsRR26 (Fig. 2d).

As OsRRs-bait constructs of type-B response regulators (except that of *OsRR21*) showed self-activation, we could not test their interactions using reciprocal combinations of bait and prey plasmids. However, we could test reciprocal combination of *OsHPTs*-prey constructs with type-A response regulators and *OsRR21*-bait constructs (Supplementary Fig. S2a–e). We couldn’t find any interaction for these reciprocal combinations.

### Determining the interactions between type-A and type-B OsRRs

**Figure 3:**
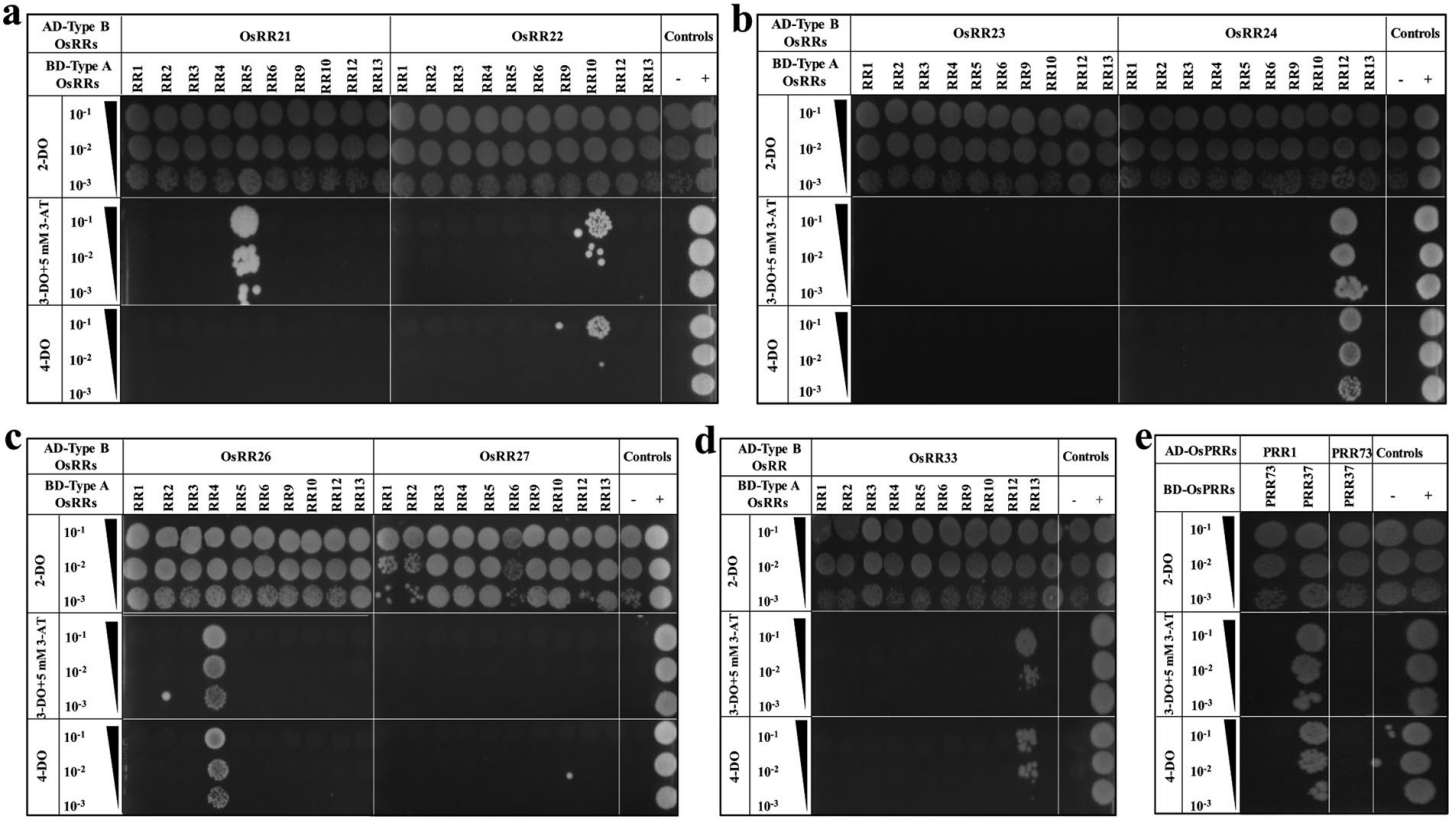
Protein–protein interaction studies among the RRs of two-component signaling system in rice. (**a**–**d**) Y2H analysis for assessment of interactions between BD-type-A and AD-type-B OsRRs and (**e**) Y2H analysis for assessment of interactions within OsPRRs. These interactions were determined using combinations of bait and prey constructs which were co-transformed into yeast. Transformants were checked for *HIS3* and *ADE2* reporter gene activation through serial dilution assays. For this, transformants were spotted on double drop-out medium (2-DO) for growth control, triple drop-out medium (3-DO) supplemented with 5 mM 3-AT) to check the activation of *HIS3* reporter gene and on quadruple drop-out medium (4-DO) to check activation of *ADE2* reporter gene. Growth on synthetically deficient triple-drop out and quadruple drop-out media indicates interaction. 10^−1^, 10^−2^ and 10^−3^ represents 10, 100 and 1,000-fold dilutions of cultures of yeast double transformants respectively. “−” and “+” signs represent negative control (host cells co-transformed with empty vectors) and positive control taken as OsSRO1a-pGAD-C1 + OsSOS1-pGBD-C1 respectively. Combinations of bait and prey constructs of TCS members co-transformed into yeast have been mentioned above the serial dilution BD-bait; AD-prey.

Combinations of type-B OsRRs as prey and type-A OsRRs as bait were co-transformed into yeast. We observed specific interactions between them (Fig. 3a–e). OsRR21 showed weak interaction with OsRR5 (Fig. 3a). OsRR22 interacted with OsRR10 (Fig. 3a). No interaction of OsRR23 was detected (Fig. 3b) whereas OsRR24 showed strong interaction with OsRR12 (Fig. 3b). OsRR26 strongly interacted with OsRR4 (Fig. 3c) while OsRR27 interactions were not detected (Fig. 3c). OsRR33 also showed strong interaction with OsRR12 (Fig. 3d).

### Determining interactions between pseudo-response regulators

To determine interactions within OsPRRs, combinations of OsPRR1-bait with OsPRR73/37-prey and OsPRR73-bait with OsPRR37-prey were checked and only one interaction was detected (Fig. 3e). OsPRR1 showed strong interaction with OsPRR37.

After determining interactions between various TCS members by examining the expression of *HIS3* and *ADE2* reporter genes by serial dilution assays, we also checked the activation of *LacZ* reporter for all the above mentioned pair-wise interactions by means of β-gal assays (filter lift assays) using X-gal as substrate. Appearance of blue colour was observed in all 24 interactions as detected by serial dilution assays. A representative β-gal assay for interaction analysis of OsPHP3 has been shown in Supplementary Fig. S3.

### *In planta* interactions of TCS proteins as observed in bimolecular fluorescence complementation (BiFC) assay

**Figure 4:**
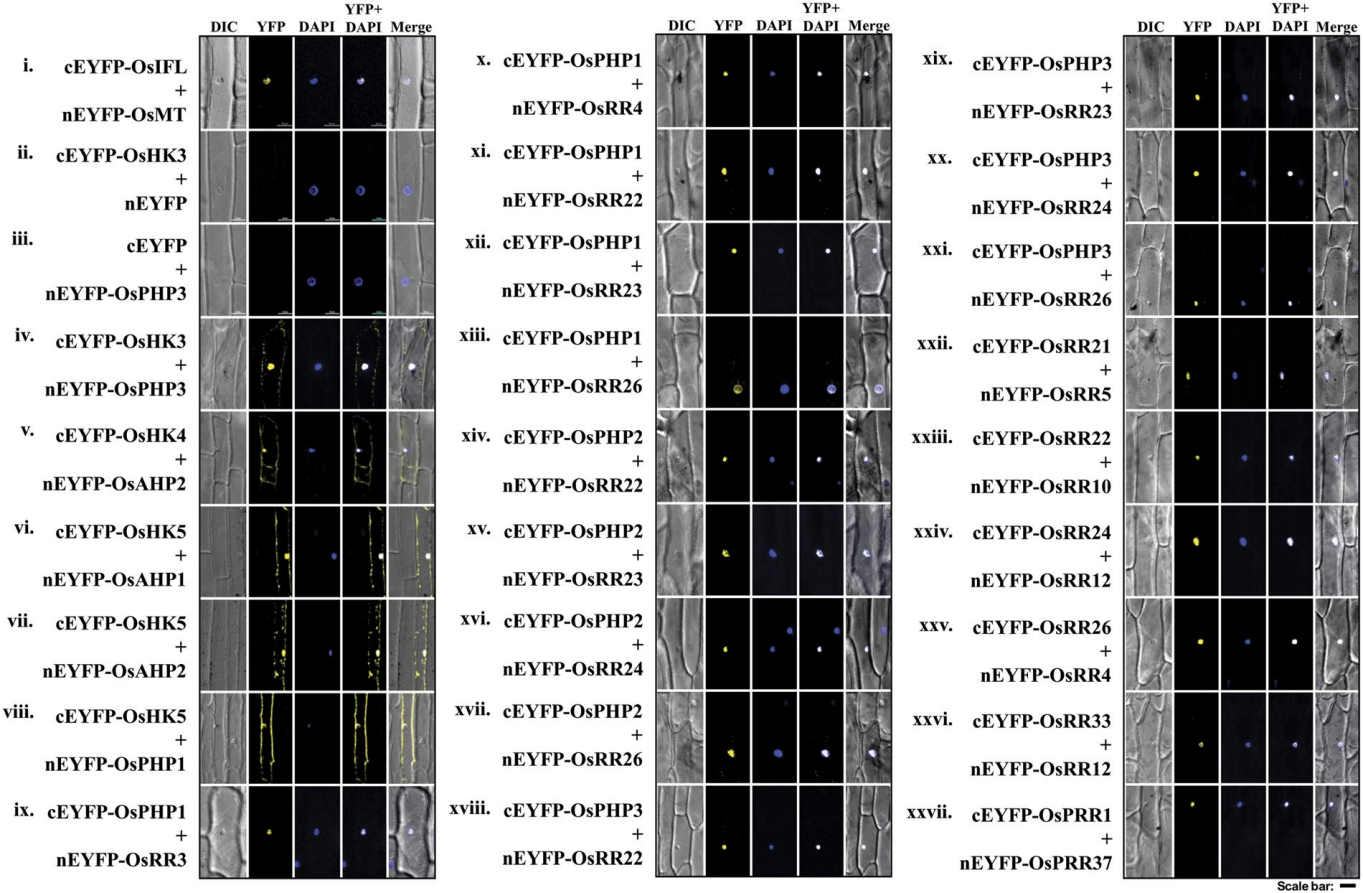
Confirmation of interactions between members of TCS in rice, using *in planta* bimolecular fluorescence complementation (BiFC) assays. Onion peel epidermal cells were co-transformed using a combination of constructs expressing proteins fused with the N (nEYFP) and C (cEYFP) termini of enhanced YFP (EYFP). Co-transformation of cEYFP-OsIF (Intermediate filaments) with nEYFP-OsMT (metallothionein) as positive control (i), cEYFP-OsHK3 with nEYFP (empty vector) and nEYFP (empty vector) with cEYFP-OsPHP3 as negative controls (ii–iii), cEYFP-OsHKs with nEYFP-OsHPTs (iv–viii); cEYFP-OsHPTs with nEYFP-OsRRs (ix–xxi); cEYFP-OsRRs (Type B) with nEYFP-OsRRs (Type A) (xxii–xxvi) and cEYFP-OsPRR1 with nYFP-OsPRR37 (xxvii) as indicated. Yellow color indicates YFP fluorescence and blue color indicates nuclei stained with DAPI; the merged image is a digital merge of bright field, DAPI and fluorescent images. Scale bar = 50 µm. BiFC assays also reveal sub-cellular localization of interacting proteins.

Yellow fluorescence confirmed interactions in OsHK3 (cytosolic fragment)-OsPHP3 pair (Fig. 4iv); OsHK4 (cytosolic fragment)-OsAHP2 pair (Fig. 4v) and OsHK5 (cytosolic fragment) with OsAHP1, OsAHP2 and OsPHP1 (Fig. 4vi–viii). These interactions were observed in the cytosol as well as in the nucleus. Furthermore, interactions of OsPHP1 with OsRR3, OsRR4, OsRR22, OsRR23 and OsRR26 were also confirmed (Fig. 4ix–xiii). Interactions of OsPHP2 (Fig. 4xiv–xvii) and OsPHP3 (Fig. 4xviii–xxi) with same set of response regulators- OsRR22, OsRR23, OsRR24 and OsRR26 were also revalidated by BiFC assays.

**Figure 5:**
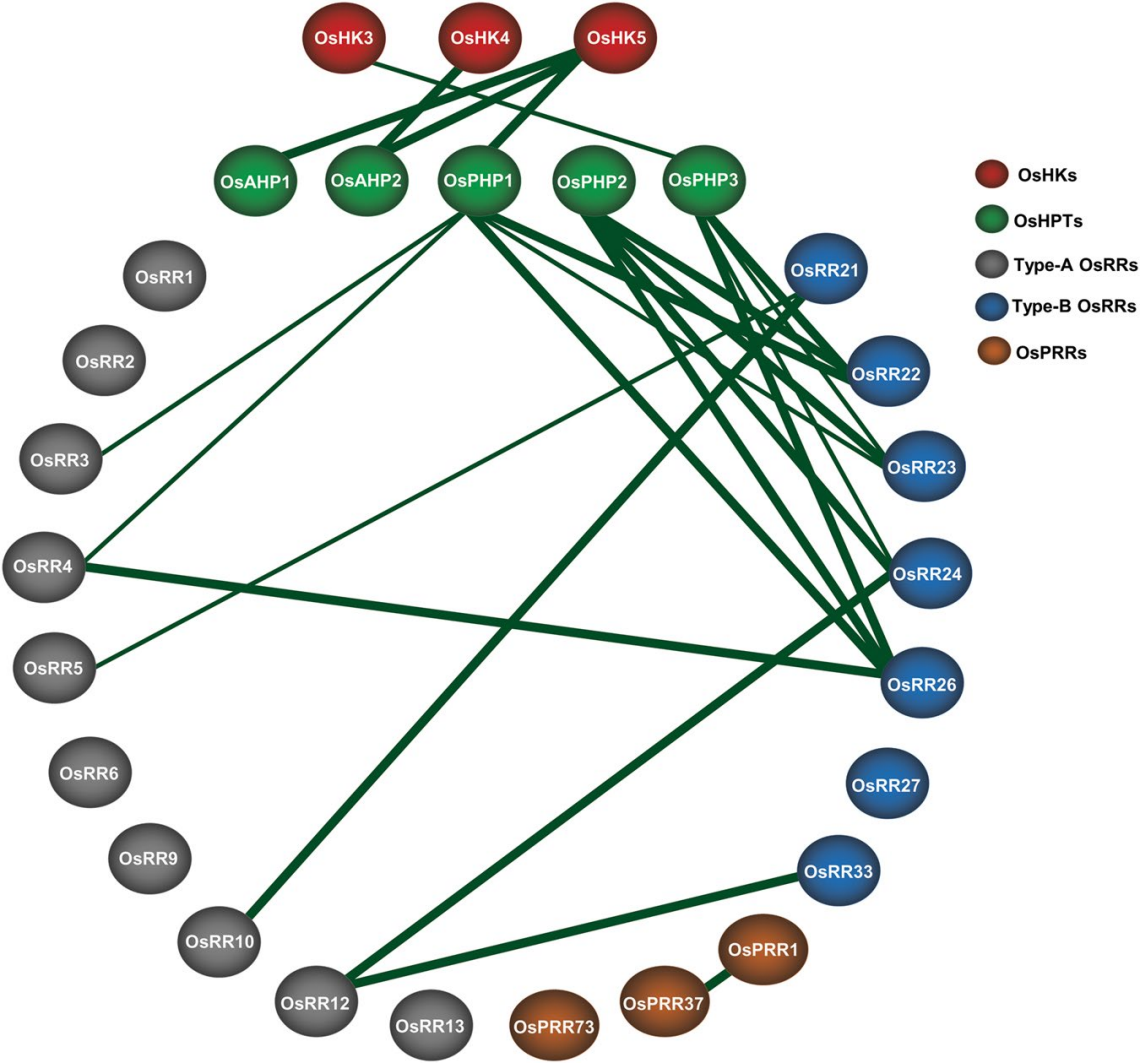
Cartoon depicting the two-component signaling proteins interactome in rice based on this study. Green lines show interactions found in the Y2H analysis and reconfirmed by BiFC assays. Thick and thin lines indicate the strong and weak protein-protein interactions respectively, as detected in Y2H study.

Unique pair-wise interactions between type-A and type-B response regulators such as OsRR21-OsRR5 (Fig. 4xxii), OsRR22-OsRR10 (Fig. 4xxiii), OsRR24-OsRR12 (Fig. 4xxiv), OsRR26-OsRR4 (Fig. 4xxv) and OsRR33-OsRR12 (Fig. 4xxvi), were also revalidated. Interaction of OsPRR1 with OsPRR37 was also confirmed (Fig. 4xxvii). Fluorescence could be detected in the nucleus, thereby indicating that interactions of OsHPTs with OsRRs, those between type-A and type-B OsRRs and of OsPRR1 with OsPRR37 take place in the nucleus. Some of the interacting partners were tested in reciprocal combinations also and were found to interact in a similar pattern as seen before in Y2H assay (Supplementary Fig. S4). Figure 5 summarizes the results of these interactions as confirmed by both the techniques. As can be seen, some of the interactions are strong (represented as thick lines) while some are weak (represented as thin lines).

## Discussion

Plants, since sessile in nature, are constantly exposed to variables and extremities in their environment, be in terms of biotic or abiotic factors^39^. Each plant species has evolved its own unique intricate machinery which perceives and responds to a given stimuli^2^. However, what lies in between the ‘perception of the stimuli’ and ‘the response’, determines the survival of a plant under a given set of conditions^40^. With the availability of complete refined genome sequences of diverse plant genera, it is now possible to look into specific gene families and discuss the roles of the members of these families in a given response^20, 41^. One can even predict the protein-protein interactions (PPI) based on their co-expression analysis^42^. However, PPI network based on actual expression of proteins in yeast system (Y2H system) and/or further substantiated by microscopic evidence (*in planta* BiFC system) is certainly more robust and reliable. Nonetheless, construction of a PPI network is always useful for not only providing clues for dissecting out the signaling pathways but also helps in assigning new functions to ‘orphan’ members of a protein family. In the present study, we have examined protein-protein interactions between OsHKs-OsHPTs; OsHPTs-OsRRs; within OsRRs and within OsPRRs using a pairwise Y2H analysis as well as BiFC assays.

We have reported earlier that TCS system of rice shows similar architecture with that of *Arabidopsis* ^20^. It is an evolutionarily conserved signaling system. Hence, it was expected that interactome analysis of rice would reveal some degree of conservation with that of *Arabidopsis*. In fact, our data indicate similar flow of signals i.e. from HKs to HPTs then to RRs while the PRRs interact among themselves. The unique interactions between type-A and type-B response regulators in rice (documented in this study) have not been reported in *Arabidopsis*. It appears that during evolution, number of TCS members and their interactions have increased in monocots in comparison to dicots. As monocots are more diverse and advanced than the dicots from evolutionary point of view, specific interactions have also evolved. Using Y2H system, we found unique interactions in 24 combinations of proteins tested, all of which are previously unknown. In our study, TCS members were expressed in both orientations as bait and prey fusions. Out of the 24 interactions, 18 could be tested in one direction only, due to self-activation shown by some members of TCS while 6 could be tested in both orientations. But as mentioned earlier, these 6 interactions were identified in only one direction. Here, it is important to mention that Y2H technique has its own limitations. In some cases, it is unable to detect weak or transient interactions. For detection of interaction, the two fusion proteins should be expressed and folded properly into a functional structure in yeast. This may be the reason why we did not observe positive interactions in a few reciprocal combinations. It is also essential that two fusion proteins should get localized into the nucleus of the yeast cell, where they can activate the reporter gene. This is the reason why we used only cytosolic parts of membranous histidine kinases of rice for their interaction analysis. Moreover, in case of a positive interaction, full length GAL4 transcription factor should also be re-constituted. Any steric hindrance due to three dimensional structures of fusion proteins, preventing this reconstitution leads to false negative results.

In Y2H analysis, self-activation shown by some TCS members indicates their direct or indirect role in transcription activation. Self-activation by type-B OsRRs confirm their ability to recruit RNA polymerase at the promoter of the reporter gene even in heterologous yeast system. Consistent with our results, type-B response regulators have been reported to show strong self-activation in other plants^43–45^. Interestingly, OsRR21 did not show self-activation though it contains receiver and Myb like DNA-binding domains, characteristics of type-B response regulators. OsRR21 might interact with other proteins having activation domain as on its own does not show this activity. Self-activation by OsAHPs probably resulted from interaction with yeast proteins containing activation domain which results into RNA polymerase recruitment at GAL4 promoter, downstream of which reporter genes are present. AHPs in other plants have also been reported to exhibit self-activation^43^. AtAHP1 has been shown to exhibit *in vivo* ability to complement a mutational lesion of the *YPD1* (HPT) gene in yeast Complementation of yeast mutants of TCS members by plant orthologs support our argument of possible interaction of OsAHPs with yeast RRs and thereby their auto-activation activity in yeast.

Although many medium and large-scale protein-protein interaction studies rely only on Y2H data^46, 47^, by conducting *in planta* BiFC assays we revalidated our results obtained from Y2H assays. All of the interactions detected in Y2H assays were re-confirmed by BiFC assays. It demonstrates the high quality and stringency of the Y2H used in this study.

Considering their biological significance, the interacting proteins exhibit co-localization in same subcellular compartment. Moreover, most of the interactions include HPTs, which shuttles between the nucleus and the cytoplasm and hence can approach to most of the other TCS members^11^ and can act as interaction hubs. Because of the same reason, interactions involving HPTs were observed in BiFC assays to take place in cytoplasm or nucleus or both. In fact, OsAhp1, OsAhp2 and OsPhp1 have been reported to be localized to both cytoplasm and nucleus. OsRr22, OsRr23 and OsRr33 are nuclear localized proteins^48^. For remaining OsHPTs and OsRRs (except for above mentioned few OsHPTs and OsRRs), the subcellular localization is not known currently. Therefore, further *in-vivo* co-localization studies are required.

Plant TCS seems to have functional redundancy^9, 12, 13, 49^ as an important and inherent characteristic. They integrate extrinsic and intrinsic signals to control various processes. Whereas such redundancy is believed to be a rare event^50^ in case of bacterial TCS, such as in *E. coli*, which has almost equal number of receptor kinases and RRs. Specificity in TCS of bacteria has been found to be very high^51^ as demonstrated with the help of large-scale phosphorelay experiments. Functional redundancy can provide a cellular architecture to incorporate divergent signals to the TCS pathway, having similar output. For example, different extrinsic factors such as the availability of phosphate^52^, circadian rhythm^53^, cold stress^54^ and intrinsic developmental processes^55, 56^ harmonize the expression of TCS genes. Finally, these divergent input signals may converge on a common output pathway, at least in part i.e. growth controlled by cytokinin^57, 58^. Functional redundancy can also be helpful in taking care of loss of function of a gene, caused by mutations. The HPTs may behave as signal integrators in TCS system as they are found to be the interaction hub. From these data, we can conclude that expansion of a prokaryotic signaling system in origin^59^, might have been adapted to function as a signal collector in eukaryotic organisms, and downstream responses have become less specific.

Our results confirm the selective interactions between OsHKs and OsHPTs. We used the cytoplasmic regions of OsHKs for interaction analysis. It has been reported in *Arabidopsis* and yeast that the cytoplasmic part, even the receiver domain of cytoplasmic part of hybrid type histidine kinases, is sufficient and necessary for interaction with HPTs^60^. It was again proved in our study as we could detect interactions of OsHKs using their cytoplasmic parts including receiver domain.

In rice, we detected interactions of OsHKs with OsAHPs as well as OsPHPs. In *A. thaliana*, histidine kinases have been demonstrated to interact with authentic phosphotransfer proteins^11^ while their interaction with pseudo phosphotransfer proteins is not reported yet although a pseudo-phosphotransfer protein, AtAHP6 has been found in *A. thaliana* which inhibits cytokinin signaling^61^. The function of OsPHPs in rice is yet to be investigated. Their interaction with both histidine kinases and response regulators is an indication of their possible role in regulation of signaling through TCS pathway. The possibility of signaling through non-orthologous HPTs in rice, however, can’t be ruled out. Moreover, in *A. thaliana*, AtAHPs have been demonstrated to interact with multiple histidine kinases^11, 43, 62^. Same has been found to be true in case of rice as in our study, we observed interactions of OsAHP2 with two OsHKs (OsHK4 and OsHK5). This indicates that different signaling pathways can share same HPTs. In rice, interaction of OsAHP2 with two histidine kinases also indicate a cross-talk between signaling mediated by different HKs, as also observed in *Arabidopsis*. Another interesting finding is that OsHK5 interact with multiple OsHPTs suggesting that multiple HPTs may be the potential downstream targets for one HK. We observed interactions of OsHPTs with both, type-A and type-B response regulators. In *Arabidopsis*, AtAHP5 has been reported to interact with both, type-A and type-B response regulators whereas AtAHP2 interacts with the type-B response regulators only^43, 62^. Similarly, in rice, OsPHP1 interacts with both type-A and type-B response regulators whereas other HPTs, such as OsPHP2 and OsPHP3, interact only with type-B OsRRs in rice. Type-B response regulators interacting with AtAHP2 in *Arabidopsis* have been shown to play a pivotal role in the response to cytokinin^9, 36, 63^. In our study, type-B OsRRs interacting with OsPHP3, also exhibit interactions with OsPHP1 and OsPHP2. OsPHP1 is the OsHPT which interacts with OsHK5. OsHK5 contains CHASE domain and its *Arabidopsis* ortholog is the ER localized cytokinin receptor. Our results suggest cross-talk between different histidine kinases. These facts also prove functional redundancy of the TCS system, but it also questions the specificity of the signal achieved and the specificity of the responses in accordance. Perhaps the same molecules may perform different functions depending upon the condition or specific signals can be a result of specific interactions between TCS members and rest of the proteome.

Type-B response regulators are transcriptional activators of type-A response regulators which in turn act as negative regulators of cytokinin signaling. The mechanism by which type-A response regulators exerts negative regulation is still not completely understood in plants. It is believed that the type-A response regulators compete with type-B OsRRs for the phosphoryl group of the activated HPTs or the interaction of type-A OsRRs with HPTs prevents them from interacting with other proteins. In the present study, we found interactions between following pairs of type-B and type-A OsRRs:- OsRR21-OsRR5; OsRR22-OsRR10; OsRR24-OsRR12; OsRR26-OsRR4 and OsRR33-OsRR12. In *Arabidopsis*, interactions between these two types of response regulators have not yet been reported^43^. We also detected interactions of OsHPTs with both types of OsRRs such as those of OsPHP1 with OsRR3, OsRR4, OsRR22, OsRR23 and OsRR26. These results are quite interesting with respect to the negative feedback regulation by type-A OsRRs. These results suggest a possible role for their direct interaction^64, 65^ and thereby probable inhibition of the type-B OsRRs by the type-A OsRRs. However, this hypothesis needs to be further validated. In *Arabidopsis*, pseudo-response regulators have been found to interact with each other. We also detected interaction between OsPRR1 and OsPRR37 in rice. Consistent with this, *Arabidopsis* ortholog AtPRR1 and AtPRR3 also interact with each other. AtPRR3 hinders ZTL-dependent degradation of TOC1/AtPRR1 and modulate its stability^66^.

BiFC assays confirmed all the interactions observed by Y2H assays. Interestingly, different interaction combinations displayed a fluorescence signal from different subcellular localizations. In rice, it has been shown that OsAHP1, OsAHP2 and OsPHP1 are localized in the nucleus and the cytosol, whereas OsRR22, OsRR23 and OsRR33 exhibit tight nuclear localization^48^. Localization of OsRR26 is not reported but it is a type-B OsRR and its *Arabidopsis* ortholog AtARR11 has been shown to be nuclear localized^67^. These facts support the nuclear interaction of OsPHP3/OsRR22 and OsPHP3/OsRR26. The nuclear interaction of OsHK-OsPHP is un-expected. Although cytosolic part of OsHKs was used for the interaction study in both Y2H and BiFC assays, it was likely to take place in the cytoplasm. Probably, cytoplasmic fragments of histidine kinases (free from being anchored into plasma membrane) get co-localised and distributed along with the corresponding interacting OsHPTs which shuttle between cytoplasm and nucleoplasm as driven by the strength of interaction.

Reports on plant TCS have not revealed any interactions between the HKs and Type-B RRs. Morever, HKs are membrane localized and Type-B RRs have been reported to be nuclear localized, rendering the physical interaction of the two highly improbable. Nevertheless, there is a probability of cytoplasmic HK fragments entering the nucleus and interacting with RRs, resulting in false positives. In addition, the major drawback of using a constitutive promoter for BiFC analysis is that the amount of protein synthesized is such that the possibility of random interactions is quite high^68^. To rule out false positives because of random interaction of TCS members due to high expression within the cell, BiFC assay for interaction between OsHK4 and OsRR26, which theoretically should not interact, was performed (Supplementary Fig. S5). As expected, no interaction was observed, which further validated the observed results were not false positives. Moreover, the probability of false positives in the BiFC assay was minimized as confocal microscopy for all interactions was performed 12 hours post transfection of the onion epidermal cells, which is much earlier than the 1 day timeframe prescribed by Xing and colleagues (2016) for minimizing false positives and considerably earlier than the 3 days required for the CaMV35S promoter to reach maximum expression^69^.

## Conclusion

Taken together, above results constitute strong arguments in favour of a cross-talk in signaling mediated by different sensory histidine kinases and function of OsPHPs as interaction hubs in rice (Fig. 5). This study revealed several possible signal movements in the form of His to Asp phosphorelay between two-component members, by all-inclusive demonstration of their physical interactions. The interactome map thus developed for rice can be compared with reported interactomes from *Arabidopsis* and populus^43, 44, 70, 71^. Our study also identified several novel potential interactions not yet reported in rice and thus may help unveil the biological roles of these proteins.

## Materials and Methods

### Media

YPAD (10 g/L yeast extract, 20 g/L peptone, 0.2 g/L Adenine, 20 g/L glucose, pH 5.8) medium and minimal synthetic drop-out media {6.7 g/L yeast nitrogen base without amino acids, 0.6 g/L 4-DO medium, 20 g/L glucose, pH 5.8, supplemented with required amino acid (0.2 g/L adenine, 0.2 g/L histidine, 0.2 g/L tryptophan, 1 g/L leucine)} were used for yeast culture. Half Murashige and Skoog (MS) medium^72^ supplemented with 3% sucrose was used in BiFC assays for incubation of onion peels.

### Host strains

AH109 strain of *Saccharomyces cerevisiae* was used for the Y2H analysis. This strain contains four reporter genes – *lacZ, MEL1, HIS3* and *ADE2*.

### Vectors

For Y2H assay, yeast expression vectors pGAD-C1 and pGBD-C1^73^ were used in this study. These vectors have Amp^R^ gene as bacterial selection marker. pGAD-C1 and pGBD-C1 vectors have coding sequence of GAL4 activation domain and GAL4 binding domain respectively, under ADH1 constitutive promoter of yeast. pGAD-C1 and pGBD-C1 contain *LEU2* and *TRP1* as yeast selection markers respectively.

For BiFC assay, pSAT1-nEYFP-C1 and pSAT1-cEYFP-C1-B vectors were used^74^. Both of these contain Amp^R^ gene as bacterial selection marker. pSAT1-nEYFP-C1 and pSAT1-cEYFP-C1-B contain N-terminal (YFP^N^) and C-terminal (YFP^C^) fragments of yellow fluorescent protein respectively, under 2XCaMV35S promoter.

### Construct preparation for Y2H assay

We attempted to amplify cDNAs encoding all non-ethylene OsHKs, OsHPTs, OsRRs (type-A, type-B and pseudo-response regulators; Table 1). Out of them, we succeeded in amplification and cloning of 3 *OsHKs*, all 5 *OsHPTs*, 10 type-A *OsRRs*, 7 type-B *OsRRs* and 3 *OsPRRs* (Table 1). For construction of yeast expression plasmids, full length genes of *OsHPTs* (*OsAHP1, OsAHP2, OsPHP1, OsPHP2, OsPHP3*), type-A *OsRRs* (*OsRR1, OsRR2, OsRR4 OsRR5, OsRR6, OsRR9, OsRR10, OsRR12, OsRR13*), type-B *OsRRs* (*OsRR21, OsRR22 OsRR23, OsRR24, OsRR26, OsRR27, OsRR33*) and pseudo-response regulators *OsPRRs* (*OsPRR1, OsPRR37, OsPRR73*) were amplified by PCR with oligonucleotide primers containing appropriate restriction sites at the ends of primers (list provided in Table 2). In case of histidine kinases (*OsHK3*, *OsHK4 and OsHK5*), cDNAs corresponding to their cytosolic parts only, were amplified and cloned because full length OsHKs are membranous proteins which cannot be used for Y2H analysis. The PCR-amplified fragments were digested with appropriate restriction enzymes and then purified from an agarose gel using Qiagen gel elution kit (Qiagen, Germany). Resulting fragments were fused “in-frame” with the coding region of the GAL4 DNA binding domain in bait vector, pGBD-C1 and of the GAL4 activation domain in prey vector, pGAD-C1. Restriction digestion and PCR reactions using vector specific primers were carried out for confirmation of cloning. All clones were re-confirmed by sequencing using vector specific primers listed in Table 2.

**Table 2:** List of primers used for Y2H analysis.

Gene	Primer	Nucleotide sequence (5′-3′)
*OsHK3* (cytosolic)	OsHK3*Sma*IF	TCCCCCGGGAAGATGAGCGAACTCAAGAAG
	OsHK3*Bam*HIR	CGGGATCCCTATTCAACTTGGTCATGATTTTG
*OsHK4* (cytosolic)	OsHK4*Eco*RIF	CGGAATTCATGTTGCTAATCGAGAGTGATTC
	OsHK4*Sal*IR	ACGCGTCGACTCAGCTGGAAACGCATGGGC
*OsHK5* (cytosolic)	OsHK5*Bam*HIF	CGGGATCCATGAGTTATGAGAGTGGATTTC
	OsHK5*Sal*IR	ACGCGTCGACCTAGGTCAATGGATCTGTTGC
*OsAHP1*	OsAHP1*Sal*IF	ATGGCGGCCGCCGCGCTG
	OsAHP1*Sal*IR	ACGCGTCGACTTAATGTTTAGGGTAACAAGCTTG
*OsAHP2*	OsAHP2*Eco*RIF	CGGAATTCATGGCGGCCGCCGCTCTC
	OsAHP2*Bam*HIR	CGGGATCCTTATTGCTGCTTGGGATCATAAG
*OsPHP1*	OsPHP1*Eco*RIF	CGGAATTCATGGATTATTCTAATTTGCGTC
	OsPHP1*Sal*IR	ACGCGTCGACTTACATGACAGGCCTAGTGG
*OsPHP2*	OsPHP2*Eco*RIF	CGGAATTCATGGAGTATTCAAATTTGCGTCG
	OsPHP2*Sal*IR	ACGCGTCGACTTACTTCCTTGAGCTCACTGC
*OsPHP3*	OsPHP2*Eco*RIF	CGGAATTCATGGAGTACGGTAATTTGCGAC
	OsPHP2*Sal*IR	ACGCGTCGACTTACTTGCCCGCAGGCCTAG
*OsPRR1*	OsPRR1*Eco*RIF	CGGAATTCATGGTGGGCGCCGGCGAG
	OsPRR1*Sal*IR	ACGCGTCGACCTACTCTGGAGAAGAAACCATC
*OsPRR37*	OsPRR37*Eco*RIF	CGGAATTCATGATGGGAACCGCTCATCA
	OsPRR37*Sal*IR	ACGCGTCGACTCATCTGTCCGCTGCCGC
*OsPRR73*	OsPRR73*Eco*RIF	CGGAATTCATGGGTAGCGCCTGCGAAG
	OsPRR73*Sal*IR	ACGCGTCGACTTAGGACTCATGACTTTGATAG
*OsRR1*	OsRR1*Eco*RIF	CGGAATTCATGGAAGGAGGAAGGGGGG
	OsRR1*Bgl*IIR	GGAAGATCTTCAAGCACACCACAGGTTGAG
*OsRR2*	OsRR2*Eco*RIF	CGGAATTCATGGGAGCGGAGGCGGTG
	OsRR2*Sal*IR	ACGCGTCGACTCATGCGCACCACAGGGAG
*OsRR3*	OsRR3*Bam*HIF	CGGGATCCATGTCGACGAAGACAGTGCC
	OsRR3*Bgl*IIR	GGAAGATCTTCATTTCATGATGACGCGGTTG
*OsRR4*	OsRR4*Eco*RIF	CGGAATTCATGACGGTGGTTGATGCGG
	OsRR4*Sal*IR	ACGCGTCGACTCAGGTCTCCACTGCAAGG
*OsRR5*	OsRR5*Eco*RIF	CGGAATTCATGGCCACCTGCAGGAGC
	OsRR5*Sal*IR	ACGCGTCGACTCACCGGAGGACGCGGC
*OsRR6*	OsRR6*Eco*RIF	CGGAATTCATGGCGGCAGCGGCGCAG
	OsRR6*Sal*IR	ACGCGTCGACTCATCTGATACGGCTGCAGAG
*OsRR9*	OsRR9*Eco*RIF	CGGAATTCATGGCAGTGGCTATAGAGGC
	OsRR9*Sal*IR	ACGCGTCGACTCAACTATGCCTTGGTCTTATTG
*OsRR10*	OsRR10*Eco*RIF	CGGAATTCATGGCAGTGGCTATAGAGGC
	OsRR10*Sal*IR	CACGCGTCGACTCAACTATGCCTTGGTCTTATTG
*OsRR12*	OsRR12*Eco*RIF	CGGAATTCATGTCATCCCCCCATGTGC
	OsRR12*Sal*IR	ACGCGTCGACTCATATGTAGTTCAGAATACGAG
*OsRR13*	OsRR13*Eco*RIF	CGGAATTCATGTCATCCCCCCATGTGC
	OsRR13*Sal*IR	ACGCGTCGACTCATATGTAGTTCAGAATACGAG
*OsRR21*	OsRR21*Eco*RIF	CGGAATTCATGGCGCCGGTGGAGGATG
	OsRR21*Sal*IR	ACGCGTCGACTCACATCTGTCCACTAAATCCG
*OsRR22*	OsRR22*Bam*HIF	CGGGATCCATGCTTCTGGGTGCTTTGAG
	OsRR22*Bgl*IIR	GGAAGATCTTCATATGCAGGCACCAAGTG
*OsRR23*	OsRR23*Eco*RIF	CGGAATTCATGAGGGCGGCGGAGGAG
	OsRR23*Bgl*IIR	GGAAGATCTTCATATGCAAGCTCCAAGGG
*OsRR24*	OsRR24*Eco*RIF	CGGAATTCATGACGGTGGAGGAGAGGC
	OsRR24*Bgl*IIR	GGAAGATCTCTAGACCAGCTCCCAGTCC
*OsRR26*	OsRR26*Eco*RIF	CGGAATTCATGGACGCCACCGCCTTC
	OsRR26*Sal*IR	CACGCGTCGACTCAGGATGATGCAAAGAGACA
*OsRR27*	OsRR27*Eco*RIF	CGGAATTCATGGCGGAGAACAACGGC
	OsRR27*Bam*HIR	CGGGATCCTCAAGGTCCACTAGATGCG
*OsRR33*	OsRR33*Bam*HIF	CGGGATCCATGGATCAAGCGAGGATCTC
	OsRR1*Bgl*IIR	GGAAGATCTCTACTCGCTCCCGGCAAG
pGAD-C1 vector specific primers	pGAD-C1 F	AACTATCTATTCGATGATGAAG
	pGAD-C1 R	GATGCACAGTTGAAGTGAAC
pGBD-C1 vector specific primers	pGBD-C1 F	CATCGGAAGAGAGTAGTAAC
	pGBD-C1 R	GATGCACAGTTGAAGTGAAC

### Construct preparation for BiFC assay

TCS members which showed positive interactions in Y2H analysis were cloned in pSAT1-nEYFP-C1 and pSAT1-cEYFP-C1-B vectors containing the multiple cloning site present downstream of the EYFP fragments coding sequence. Cloning was confirmed by restriction digestion as well as PCR using vector specific primers. All constructs were sequenced to verify frame and cDNA sequence correctness. The primers used are listed in Table 3.

**Table 3:** List of primers used for BiFC assays.

Gene	Primer	Nucleotide sequence (5′-3′)
*OsHK3* (cytosolic)	OsHK3BiFC*Sma*IF	CCCCCGGGATGAGCGAACTCAAGAAG
	OsHK3BiFC*Bam*HIR	CGGGATCCCTATTCAACTTGGTCATG
*OsHK4* (cytosolic)	OsHK4BiFC*Sal*IF	ACGCGTCGACATGGATTGCCGGAAAATGGAAGCG
	OsHK4BiFC*Bam*HIR	CGGGATCCTCAGCTGGAAACGCATGGGC
*OsHK5* (cytosolic)	OsHK5BiFC*Sal*IF	ACGCGTCGACATGGAAGAGGCAGAAGATAATTATACG
	OsHK5BiFC*Bam*HIR	CGGGATCCTTAAGCACATGGCTGAAGGCGT
*OsPHP1*	OsPHP1BiFC*Eco*RIF	CGGAATTCAATGGATTATTCTAATTTGCGTC
	OsPHP1BiFC*Sal*IR	ACGCGTCGACTTACATGACAGGCCTAGTGG
*OsPHP2*	OsPHP2BiFC*Eco*RIF	CGGAATTCAATGGAGTATTCAAATTTGCGTCG
	OsPHP2BiFC*Sal*IR	ACGCGTCGACCCTTCCTTGAGCTCACTGCATA
*OsPHP3*	OsPHP3BiFC*Eco*RIF	CGGAATTCAATGGAGTACGGTAATTTGCGAC
	OsPHP3BiFC*Sal*IR	ACGCGTCGACTTACTTGCCCGCAGGCCTAG
*OsPRR1*	OsPRR1BiFC*Eco*RIF	CGGAATTCAATGGTGGGCGCCGGCGAG
	OsPRR1BiFC*Sal*IR	ACGCGTCGACCTACTCTGGAGAAGAAACCATC
*OsPRR37*	OsPRR37BiFC*Eco*RIF	CGGAATTCAATGATGGGAACCGCTCATCA
	OsPRR37BiFC*Sal*IR	ACGCGTCGACTCATCTGTCCGCTGCCGC
*OsRR3*	OsRR3BiFC*Bgl*IIF	GGAAGATCTATGTCGACGAAGACAGTGCC
	OsRR3BiFC*Bam*HIR	CGGGATCCTCATTTCATGATGACGCGGTTG
*OsRR4*	OsRR4BiFC*Eco*RIF	CGGAATTCAATGACGGTGGTTGATGCGG
	OsRR4BiFC*Sal*IR	ACGCGTCGACTCAGGTCTCCACTGCAAGG
*OsRR5*	OsRR5BiFC*Eco*RIF	CGGAATTCAATGGCCACCTGCAGGAGC
	OsRR5BiFC*Sal*IR	ACGCGTCGACTCACCGGAGGACGCGGC
*OsRR10*	OsRR10BiFC*Eco*RIF	CGGAATTCAATGGCAGTGGCTATAGAGGC
	OsRR10BiFC*Bam*HIR	CGGGATCCTCAACTATGCCTTGGTCTTATT
*OsRR12*	OsRR12BiFC*Eco*RIF	CGGAATTCAATGTCATCCCCCCATGTGC
	OsRR12BiFC*Sal*IR	ACGCGTCGACTCATATGTAGTTCAGAATACGAG
*OsRR21*	OsRR21BiFC*Eco*RIF	CGGAATTCAATGGCGCCGGTGGAGGATG
	OsRR21BiFC*Sal*IR	ACGCGTCGACTCACATCTGTCCACTAAATCCG
*OsRR22*	OsRR22BiFC*Bgl*IF	GGAAGATCTATGCTTCTGGGTGCTTTGAG
	OsRR22BiFC*Bam*HIR	CGGGATCCTCATATGCAGGCACCAAGTG
*OsRR23*	OsRR23BiFC*Bgl*IIF	GGAAGATCTATGAGGGCGGCGGAGGAG
	OsRR23BiFC*Eco*RIR	CGGAATTCTCATATGCAAGCTCCAAGGG
*OsRR24*	OsRR24BiFC*Bgl*IIF	GGAAGATCTATGACGGTGGAGGAGAGGC
	OsRR24BiFC*Bam*HIR	CGGGATCCCTAGACCAGCTCCCAGTCC
*OsRR26*	OsRR26BiFC*Eco*RIF	CGGAATTCAATGGACGCCACCGCCTTC
	OsRR26BiFC*Sal*IR	CACGCGTCGACTCAGGATGATGCAAAGAGACA
*OsRR33*	OsRR33BiFC*Bgl*IIF	GGAAGATCTATGGATCAAGCGAGGATCTC
	OsRR1BiFC*Bam*HIR	CGGGATCCCTACTCGCTCCCGGCAAG
pSAT1-cEYFP-C1-B vector specific primers	pSAT1-cEYFP-C1-B F	GTCCTGCTGGAGTTCGTGAC
	pSAT1-cEYFP-C1-B R	GAACTACTCACACATTATTCTGG
pSAT1-nEYFP-C1 vector specific primers	pSAT1-nEYFP-C1 F	CAGAAGAACGGCATCAAGGTG
	pSAT1-nEYFP-C1 R	GAACTACTCACACATTATTCTGG

### Transformation of Saccharomyces cerevisiae

30 ml of YPAD broth was inoculated with two to four yeast colonies that were freshly revived. The culture was incubated for 18–24 hours at 30 °C with shaking at 225 rpm. Competent cells were prepared and were used for transformation immediately as described earlier^75^. The plates were incubated at 30 °C for 3–5 days until colonies appeared.

### Yeast two-hybrid assay

Y2H experiments were performed using AH109 strain which was transformed with the pairs of appropriate constructs. Yeast double transformants were selected on double drop-out medium lacking leucine and tryptophan for 3–5 days at 30 °C as pGAD-C1 and pGBD-C1 vectors contain *LEU2* and *TRP1* selection marker genes respectively. Subsequently, to check potential interactions, the doubly transformed colonies were assessed for *HIS3* and *ADE2* reporters through growth on synthetically deficient triple drop out (lacking leucine, tryptophan and histidine) and quadruple drop out (lacking leucine, tryptophan, histidine and adenine) medium. Transformants were grown in double drop out liquid medium and serially diluted by 10, 100 and 1000 folds and subsequently spotted (10 µl of each dilution) on double, triple and quadruple media. Triple drop out medium was supplemented with 5 mM 3-amino-1,2,4-triazole (3-AT) which is a competitive inhibitor of the product of the *HIS3* gene, imidazole glycerol-phosphate dehydratase enzyme that catalyses one of steps of histidine biosynthesis pathway. Growth of transformants on quadruple drop-out medium shows strong interaction. Host cells co-transformed with empty vectors were taken as negative control and those co-transformed with OsSRO1a-pGAD-C1 + OsSOS1-pGBD- C1 were taken as positive control^75^.

### Filter lift assay

The transformed colonies were streaked on YPDA agar plates and incubated at 30 °C for 24–48 hrs. For the β-galactosidase assay, the colonies grown on YPDA media were transferred to Whatman 3 mm filter paper and were cracked open by freeze-thaw method using liquid nitrogen. Whatman paper was soaked in Z buffer (60 mM Na_2_HPO_4_, 40 mM NaH_2_PO_4_, 10 mM KCl, 1 mM MgSO_4_, pH 7.0) containing 0.27% β-mercaptoethanol and 0.5 mg/ml X-gal (5-bromo-4-chloro-3-indolyl-L-D-galactopyranoside). The filter paper was incubated at 30 °C in the dark for several hours, and the development of blue colour was monitored.

### Biolistic transformation of onion epidermal cells and YFP visualization

Pair wise combinations of BiFC constructs of TCS members were co-transformed in the onion epidermal peel cells by particle bombardment method as described^76^. Onion peels were mounted on microscopic slide and YFP (excitation wavelength 514 nm, emission wavelength 527 nm) fluorescence was viewed using confocal microscope. OsHK3 + nEYFP (empty vector) and cEYFP (empty vector) + OsPHP3 were used as the negative controls whereas cEYFP-OsIF + nEYFP-OsMT constructs^76^ were used as the positive control. All BiFC experiments were repeated three times.

### Data Availability Statement

All data generated or analysed during this study are included in this published article (and its Supplementary Information files).

## Electronic supplementary material

Supplementary Dataset 1
