# Supplementary material for: Mapping the ‘Two-component system’ network in rice
Source: Sci Rep. 2017 Aug 24;7:9287. doi: 10.1038/s41598-017-08076-w (PMC5571105; doi:10.1038/s41598-017-08076-w)
Supplement: Supplementary file 1 — Supplementary Dataset 1 [file 41598_2017_8076_MOESM1_ESM.doc]

*Supplementary information*

**Mapping the ‘Two-component system’ network in rice**

Ashutosh Sharan^1#^, Praveen Soni^1#^, Ramsong Chantre Nongpiur^1^, Sneh L Singla-Pareek^2^ and Ashwani Pareek^1^*

^1^Stress Physiology and Molecular Biology Laboratory; School of Life Sciences; Jawaharlal Nehru University; New Delhi, India;

^2^Plant Stress Biology; International Centre for Genetic Engineering and Biotechnology; New Delhi, India

Ashutosh Sharan

*E-mail: ashu20here@gmail.com*

Praveen Soni

*E-mail:* [*praveen.soni15@gmail.com*](mailto:praveen.soni15@gmail.com)

Ramsong Chantre Nongpiur

*E-mail:* ramsongnongpiur@gmail.com

Sneh L Singla-Pareek

*E-mail:* [*sneh@icgeb.res.in*](mailto:sneh@icgeb.res.in)

*^#^ Equal contribution*

**To whom correspondence should be addressed:*

Ashwani Pareek

Stress Physiology and Molecular Biology Laboratory,

School of Life Sciences, Jawaharlal Nehru University,

New Delhi 110067, India

Phone: +91-11-26704504

Fax No.: +91 11 26742558

E-mail: [ashwanip@mail.jnu.ac.in](mailto:ashwanip@mail.jnu.ac.in)

**Supplementary Figure S1: Self activation studies of members of the two-component signaling system of rice. (a)** For prey-constructs of TCS members, yeast transformants were grown in SD-Leu liquid medium and then serially diluted to 10, 100 and 1000 fold and subsequently spotted on SD-Leu drop medium (1-DO) for growth control, two-drop out medium (2-DO+5 mM 3-AT) supplemented with 5 mM 3-AT lacking leucine and histidine to check the activation of *HIS3* reporter gene and on three drop out medium (3-DO) lacking leucine, histidine and adenine to check activation of *ADE2* reporter gene. None of the pGAD-C1 constructs activated reporter genes. **(b)** For bait-constructs of TCS members, yeast transformants were grown in SD-Trp liquid medium and then serially diluted to 10, 100 and 1000 fold and subsequently spotted on SD-Trp drop medium (1-DO) for growth control, two-drop out medium (2-DO+5 mM3-AT) supplemented with 5 mM 3-AT lacking tryptophan and histidine to check the activation of  *HIS3* reporter gene and on three drop out medium (3-DO) lacking tryptophan, histidine and adenine to check activation of *ADE2* reporter gene. Some of the pGBD-C1 constructs (OsAHP1-2, OsRR22-24, 26, 27, 33) activated reporter genes. 10^-1^, 10^-2^ and 10^-3^ represents 10, 100 and 1000 fold dilutions of cultures of yeast transformants respectively. “-” and “+” signs represent negative control (host cells co-transformed with empty vectors) and positive control taken as OsSRO1a-pGAD-C1 + OsSOS1-pGBD-C1 respectively.

**Supplementary Figure S2: Test for interaction of OsHPTs with OsRRs in reciprocal combinations.** OsHPT-prey and OsRRs-bait constructs (in pairs) were co-transformed into AH109 strain of yeast. Transformants were assayed for *LacZ* activation as well as *HIS3 and ADE2* reporter genes assay. Interaction study of OsRRs (Type A and OsRR21) with OsPHP1-3 (**a-c**) and OsAHP1-2 (**d-e**) has been shown. No interaction was found in reciprocal combinations. **(i)** Diagrammatic representation of constructs transformed in AH108 strain of yeast. +: positive control; -: negative control **(ii)** Filter lift assay **(iii)** Streaking of colonies on three drop out (3-DO) medium lacking leucine, histidine and adenine supplemented with 5mM 3-AT. **(iv)** Streaking of colonies on four drop out (4-DO) medium lacking leucine, tryptophan, histidine and adenine.

**Supplementary Figure S3: Test for interaction of OsRRs with OsPHP3.** OsRR-prey and OsPHP3-bait constructs (in pairs) were co-transformed into AH109 strain of yeast. Transformants were assayed for *LacZ* activation as well as *HIS3 and ADE2* reporter genes assay**. (i)** Diagrammatic representation of constructs transformed in AH108 strain of yeast. +: positive control; -: negative control **(ii)** Filter lift assay **(iii)** Streaking of colonies on three drop out medium lacking leucine, histidine and adenine (3-DO) supplemented with 5mM 3-AT. **(iv)** Streaking of colonies on four drop out (4-DO) medium lacking leucine, tryptophan, histidine and adenine. OsRR22 and OsRR26 show strong interaction with OsPhp3 as revealed by the expression of the reporter genes.

**Supplementary Figure S4. Interactions between some members of TCS in reciprocal combination, using bimolecular fluorescence complementation (BiFC) assays.** Onion peel epidermal cells were co-transformed using reciprocal combination of constructs expressing proteins fused with the N (nEYFP) and C (cEYFP) termini of enhanced YFP (EYFP). Cells co-transformed with nEYFP (empty vector) + nEYFP (empty vector) were used as negative control (i), nEYFP-OsHKs with cEYFP-OsHPTs (ii-iii); nEYFP-OsPHP1 with cEYFP-OsRR26 (iv); nEYFP-OsRR24 (Type B) with cEYFP-OsRR12 (Type A) (v) as indicated. Similar result was observed in reciprocal combination as was found in normal condition (Figure 4). Yellow color indicates YFP fluorescence and blue color indicates nuclei stained with DAPI; the merged image is a digital merge of bright field, DAPI and fluorescent images. Scale bar =50 µm. BiFC assays also reveal sub-cellular localization of interacting proteins.

**Supplementary Figure 5. Confocal microscopy showing lack of interaction between OsHk4 and OsRr26 using *in planta* bimolecular fluorescence complementation (BiFC) assay.** Onion peel epidermal cells were co-transformed using a combination of constructs expressing OsHK4 fused with the N-terminal (nEYFP) and OsRR26 with C (cEYFP) terminal of enhanced YFP (EYFP) respectively. Lack of yellow color implies no YFP fluorescence, blue color indicates nuclei stained with DAPI; the merged image is a digital merge of bright field, DAPI and fluorescent images. Scale bar =50 µm.
